# Effectiveness of pulmonary rehabilitation programmes and/or respiratory muscle training in patients with post-COVID conditions: a systematic review

**DOI:** 10.1186/s12931-024-02857-4

**Published:** 2024-06-19

**Authors:** Lucía Ortiz-Ortigosa, Paula Gálvez-Álvarez, María Jesús Viñolo-Gil, Manuel Rodriguez-Huguet, Jorge Góngora-Rodríguez, Rocío Martín-Valero

**Affiliations:** 1 Department of Physiotherapy, Faculty of Health Science, University of Malaga, Málaga, 29071 Spain; 2 Department of Nursing and Physiotherapy, University of Cadiz, Cádiz, Spain

**Keywords:** post-COVID-19 condition, Sequelae, Pulmonary rehabilitation program, Respiratory muscle training

## Abstract

**Background:**

The term “post-COVID-19 condition” refers to the symptomatology that appears between four to twelve weeks after Covid-19 infection. These symptoms can persist for weeks or even months, significantly diminishing the quality of life for affected individuals. The primary objective of this study was to assess the effectiveness of pulmonary rehabilitation programs and/or respiratory muscle training on respiratory sequelae in patients with post-COVID condition.

**Methods:**

The literature search was conducted in the following databases: PubMed, PEDro, Embase, Cochrane, Scopus, and Web of Science. Randomized clinical trials were included in which participants were aged 18 years or older. Articles were excluded if at least one of the therapies did not involve pulmonary rehabilitation or respiratory muscle training, if the participants were COVID positive, if studies lacked results, and finally, if interventions were conducted without supervision or at home. This review only encompasses supervised non-virtual interventions. This study adheres to the PRISMA statement and has been registered in the PROSPERO database (CRD42023433843).

**Results:**

The outcomes obtained in the included studies are assessed across the following variables: Exercise capacity using the 6-minute walk test, Dyspnea, fatigue, Pulmonary function, Maximum inspiratory pressure, and Quality of life.

**Conclusion:**

Despite the absence of a specific treatment at present, it was evident from this review that a well-structured pulmonary rehabilitation program that incorporates both aerobic and muscular strength exercises along with techniques and inspiratory muscle exercises was the most effective form of treatment.

## Background

In 2019, a new virus emerged, the SARS-CoV-2. COVID-19 is an acute respiratory illness caused by the SARS coronavirus (SARS-CoV). Shortly thereafter, it spread worldwide, leading to a global crisis in the fields of healthcare, economy, and society [1, 2].

A significant portion of the population experienced mild symptoms, with the most commonly recurring ones being fever, non-productive cough, dyspnea, fatigue, and myalgia. However, other symptoms such as headaches, alteration or los of taste, rhinorrhea, pharyngalgia, nausea, vomiting, or diarrhea may also manifest [3, 4].

The health of individuals could be severely compromised with the emergence of more severe symptoms, such as hypoxia, respiratory failure, acute respiratory distress syndrome, and even multiorgan failure. In some cases, during the acute phase, the development of neurological complications could occur, including encephalopathies, strokes, delirium, and inflammatory syndromes of the central nervous system, among other conditions [4].

The condition that emerged between four to twelve weeks after a Covid-19 infection is commonly referred to as “post-COVID-19 condition.” These symptoms may persist for weeks or even months, significantly diminishing the quality of life for affected individuals [5, 6].

As a general rule, the most commonly recurring symptoms that persist after Covid-19 are fatigue, muscle pain, cognitive impairment, anxiety, and shortness of breath. Additionally, cardiovascular conditions and central nervous system complications can also manifest in some cases [6].

Various terms have been used to define this condition, including “long-COVID” or “persistent COVID,” “ongoing COVID,” “post-COVID syndrome,” and “post-acute COVID syndrome.” Due to the lack of consensus on a single term, the World Health Organization (WHO) has defined it as “post-COVID-19 condition” [4, 6].

Pulmonary rehabilitation programs are described as one of the primary non-pharmacological interventions for treating the sequelae of COVID-19, as they can improve respiratory function and quality of life in patients who have recovered from the coronavirus [7, 8].

The deterioration in the quality of life for the affected population, coupled with the associated disability it entailed, had a significant impact on rehabilitation units. In light of the scarcity of articles that specifically studying which treatment is most effective for our target population, compeled us to review the existing literature. This was essential to assess current treatment plans and determine which proved to be the most effective.

### Objective

The primary objective of this systematic review was to evaluate the effectiveness of pulmonary rehabilitation programs and/or respiratory muscle training on respiratory sequelae in patients with post-COVID condition.

## Methodology

This systematic review has been conducted following the recommendations of the Preferred Reporting Items for Systematic Reviews and Meta-Analysis (PRISMA) [9]. The PRISMA checklist is detailed in Anexo 1.

Furthermore, it has been registered in the International Prospective Register of Systematic Reviews (PROSPERO) with the registration number CRD42023433843.

### Search strategy

A literature review was conducted in April-May 2023, with the latest search conducted in September 2023, using the PICO framework, specifying the following:

- Population (P): Individuals with post-COVID sequelae or post-COVID-19 condition.
- Intervention (I): Pulmonary rehabilitation.
- Comparison (C): It was compared with non- intervention or conventional intervention alone or with other treatments.
- Outcome (O): The impact on various variables within the articles following the intervention.

The databases used for the search included PubMed, PEDro, Embase, Cochrane, Scopus, and Web of Science. The search terms are detailed in Table 1, and the specific search strategies for each database are provided in Appendix 2.

**Table 1:** Search strategy

Databases	Total Articles Found	Search
PubMed	650	(Breathing training) OR (Pulmonary rehabilitation) OR (Respiratory muscle training) OR (Inspiratory muscle training) OR (expiratory muscle training) OR (Maximum inspiratory pressure) OR (Maximum expiratory pressure) OR (Respiratory rehabilitation) AND (Long COVID patients) OR (Post COVID syndrome) OR (Patients with COVID sequelae).
PEDro	4	
Embase	46	
Cochrane	27	
Scopus	100	
Web of Science	175	

### Eligibility criteria

#### Inclusion criteria

Randomized clinical trials and studies involving participants over 18 years of age were included in the review.

#### Exclusion criteria

Articles in which participants were in the acute phase of the disease were excluded, as the aim was to study a population with post-COVID sequelae. Studies that did not involve pulmonary rehabilitation or respiratory muscle training as at least one of the therapies were not considered. Additionally, articles that had not completed the research or did not have results were excluded. Finally, articles where the intervention was virtual (not face-to-face) and unsupervised were also excluded.

#### Review of articles

Prior to the selection, duplicate articles were removed. Subsequently, the titles and abstracts of various studies were examined. Finally, a comprehensive analysis of the full texts of the preselected articles was conducted to verify that the selected articles met the previously mentioned inclusion and exclusion criteria. This process was carried out by three of the researchers (LOO, PGA, and JGR). Any uncertainties were resolved through consensus with another author (RMV).

### Data obtained from included studies

Detailed information was obtained from the articles, including author, year of publication, study type, interventions applied in the different study groups, observed variables, and results.

### Assessment of methodological quality and risk of bias

The assessment of methodological quality of the selected studies was carried out using the Physiotherapy Evidence Database (PEDro) scale, which measures the internal validity of the studies through 11 items [10, 11].

Regarding the risk of bias, each of the articles was assessed using the Cochrane Collaboration’s tool [12]. The following types of bias were assessed: selection bias, performance bias, detection bias, attrition bias, reporting bias, and other biases [12].

Two of the researchers (LOO and PGA) independently assessed the methodological quality. In case of any doubts or disagreements, they were resolved through consensus with another author (RMV).

Regarding the risk of bias, two of the authors (MJVG and MRH) were responsible for its assessment. In cases of doubt, it was resolved through consensus with another author (LOO).

### Data analysis

The assessment will be conducted through a qualitative analysis (narrative synthesis) to evaluate the effectiveness of pulmonary rehabilitation programs and/or respiratory muscle training. A meta-analysis was attempted with the Cochrane Collaboration’s tool of the five studies [13–17] that provide numerical data for its performance; however, given the methodological, clinical and statistical heterogeneity, it was not possible.

## Results

### Study selection

**Fig. 1:**
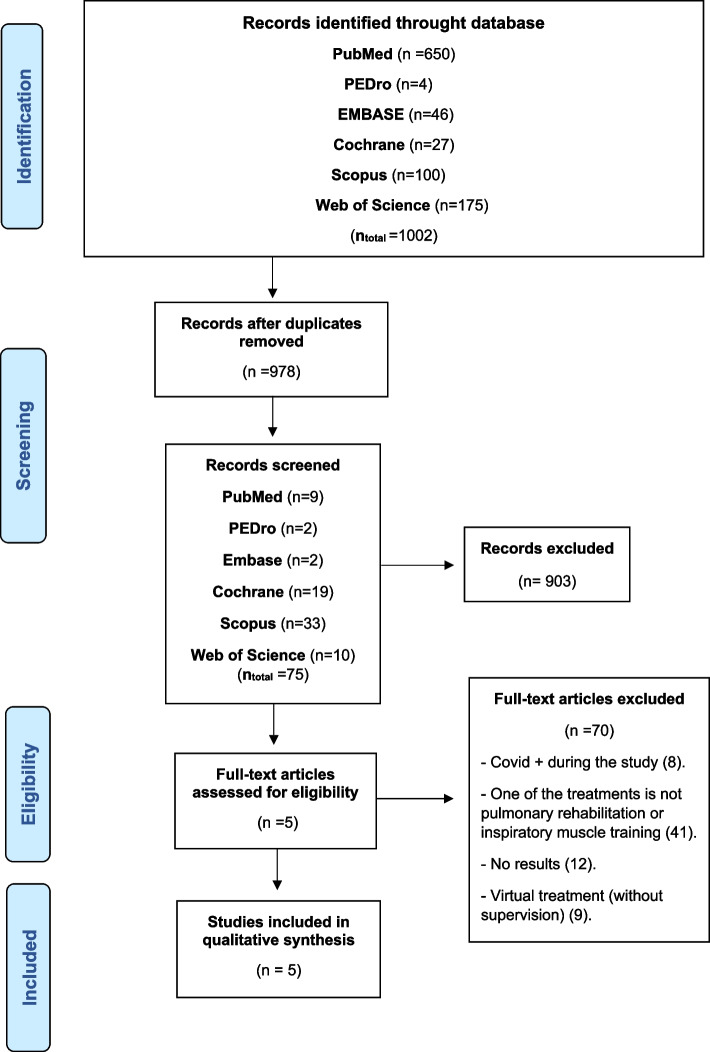
Flowchart

After the search strategies, a total of 1002 articles were found. After applying filters, the number was narrowed down to 75 studies. Upon reviewing the titles and abstracts, a total of 70 articles were excluded for not meeting the inclusion and exclusion criteria detailed earlier. Subsequently, a full reading of the articles was conducted for a more in-depth evaluation. In the end, a total of 5 studies were included. The detailed selection process for the articles included in this review can be found in Fig. 1.

*From*: Moher D, Liberati A, Tetzlaff J, Altman DG, The PRISMA Group (2009). *P*referred *R*eporting *I*tems for *S*ystematic Reviews and *M*eta-*A*nalyses: The PRISMA Statement. PLoS Med 6 (7): e1000097. 10.1371/journal.pmed1000097.

### Methodological quality of included studies

In Table 2, you can observe the scores obtained from the included studies after the methodological quality assessment conducted using the PEDro scale. According to the scale, three of the articles obtained a score of 7, categorizing them as level of evidence 1. The remaining two articles received a score of 6, indicating level of evidence 2.

**Table 2:** Methodological quality according to the PEDro scale

CRITERIA	1	2	3	4	5	6	7	8	9	10	11	TOTAL
**Nagy et al.** [13]	Y	Y	N	Y	Y	N	N	Y	Y	Y	Y	7
**Rutkowski et al.** [14]	Y	Y	Y	Y	N	N	N	Y	Y	Y	Y	7
**Alshaimaa et al.** [15]	Y	Y	N	Y	Y	N	N	Y	Y	Y	Y	7
**Jimeno et al.** [16]	Y	Y	N	Y	N	N	N	Y	Y	Y	Y	6
**Jimeno et al.** [17]	Y	Y	N	Y	N	N	N	Y	Y	Y	Y	5

The eligibility criteria do not contribute to the total score. Y: Yes; N: No

*PEDro* Physiotherapy Evidence Database

### Risk of bias of included studies

**Fig. 2:**
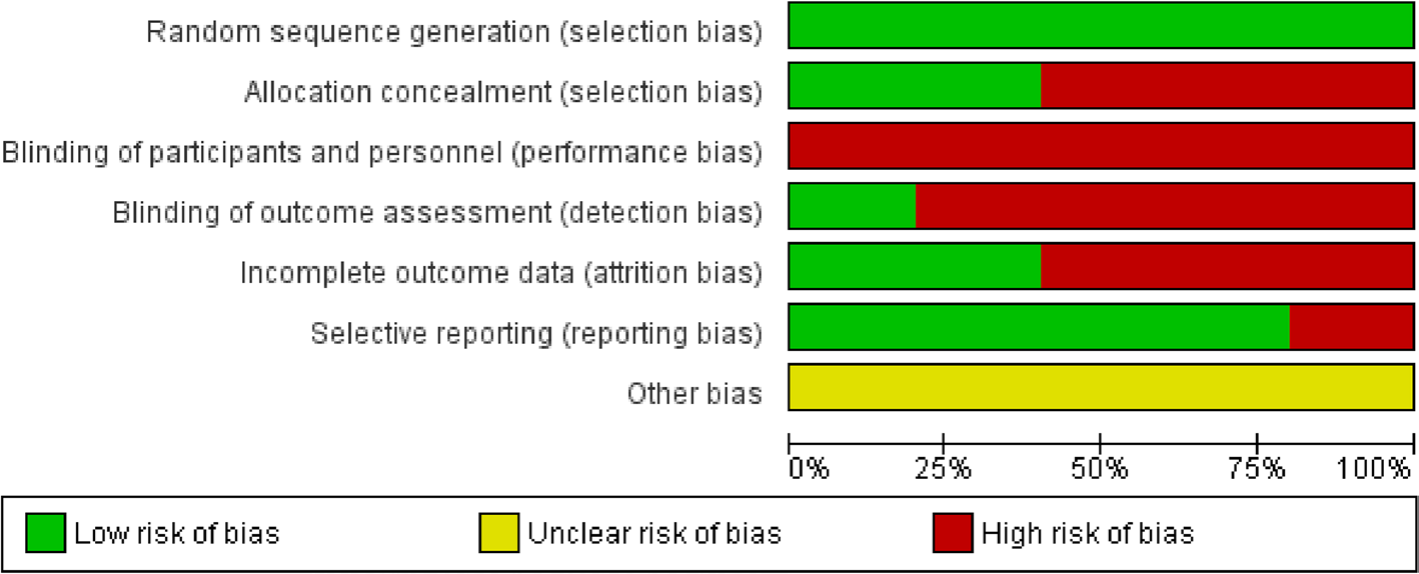
Risk of bias graph

The risk of bias of the articles that were included in this review was assessed using the Cochrane Risk of Bias Assessment Tool [18]. The results of the risk of bias assessment are shown in Fig. 2.

**Fig. 3:**
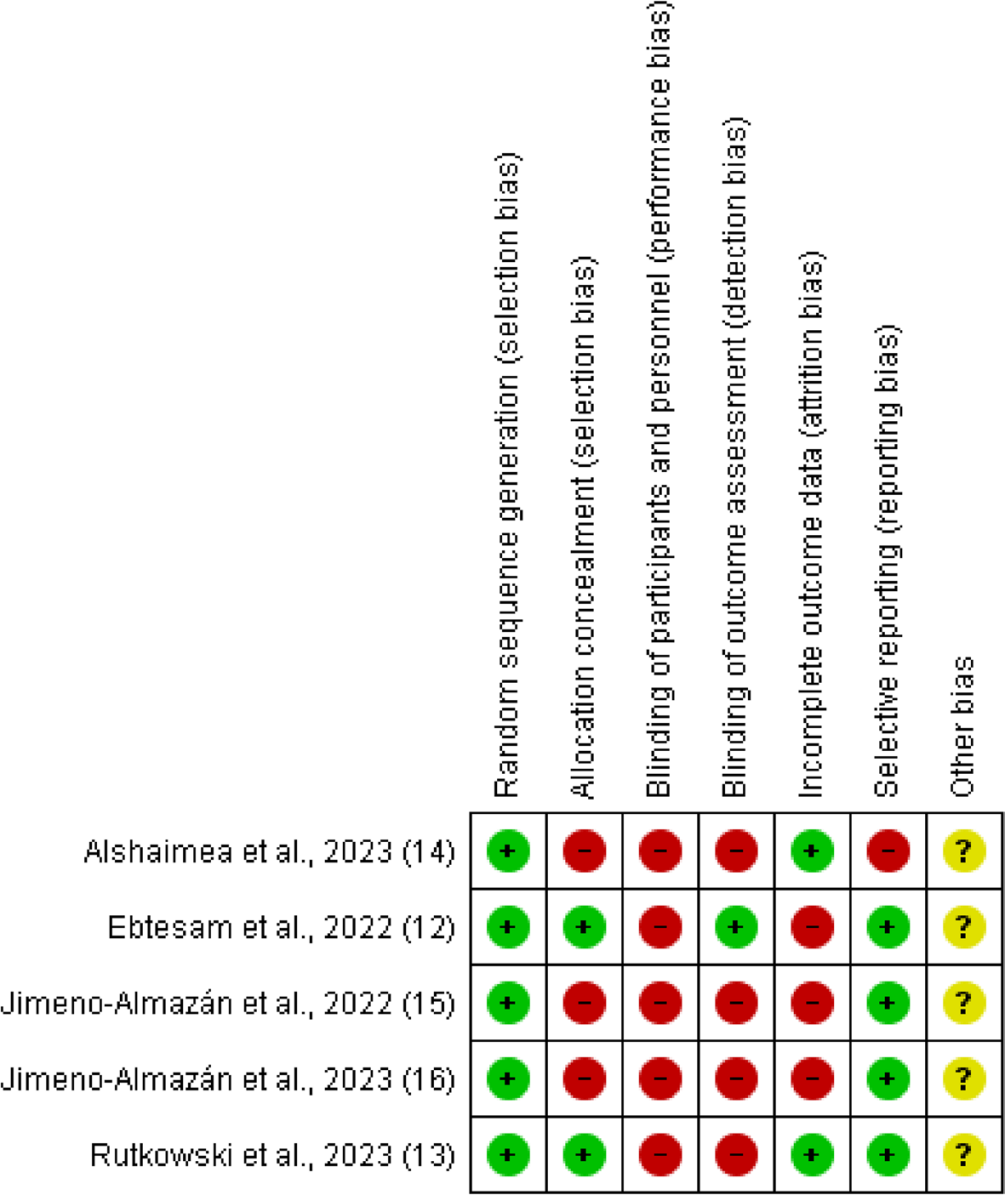
Risk of bias summary

The study by Rutkowski et al. has the lowest risk of bias and offers the most confidence in its results, while the study by Alshaimea et al. has the highest risk of bias and offers the least confidence in its results. The studies by Ebtesam et al. and Jimeno-Almazán et al. have an intermediate risk of bias and offer moderate confidence in their results (Fig. 3).

### Study design

Next, the key characteristics of the selected articles are summarized (Table 3). A total of 263 individuals participated in the selected studies. The article with the largest sample size included a total of 80 participants [17], while the study with the fewest participants involved a total of 32 individuals [14]. The age of the participants ranged from 30 to 80 years. However, in two of the articles, the age range was not specified [16, 17]. Regarding the intervention duration, the longest period was twelve weeks [15], while the shortest was three weeks [14]. The specific intervention of each article, the examined variables, and the main results are displayed in Table 3.

**Table 3:** PICO table (Its place within the text is in the “Study design” section)

Author and year	Study title	Type of study	Intervention	Variables	Results
(Nagy et al., 2022) [13]	Influence of manual diaphragm release technique combined with inspiratory muscle training on selected persistent symptoms in men with post-covid-19 syndrome: A randomized controlled trial	Randomized controlled trial. *N* = 52. Participant age: 30–45 years. Intervention time: 6 weeks	**I.G**: diaphragm release plus Inspiratory muscle training via POWERbreath. **C.G**: Inspiratory muscle training	• PImax. • SBP. • DBP. • 6-MWTD. • Fatigue: FSS. • Dyspnea: MMRC. • Serum lactate level.	The results showed a statistically significant improvement for all secondary outcomes in both groups. However, in the main outcome, maximum static inspiratory pressure, it increased significantly by 48.17% (*p* < 0.001) in the intervention group with no significant change in the control group.
(Rutkowski et al., 2023) [14]	Inpatient post-COVID-19 rehabilitation program featuring virtual reality—Preliminary results of randomized controlled trial	Preliminary results of randomized controlled trial *N* = 32. Participant age: 40–80 years. Intervention time: 3 weeks	**I.G**: pulmonary rehabilitation through virtual reality. **C.G**: traditional pulmonary rehabilitation program.	• 6-MWTD. • FEV_1_. • FVC and FEV_1_. • TLC. • Perceived dyspnea: the Borg scales. • Stress level: The Perceived Stress Scale (PSS-10).	Regarding exercise capacity, both groups improved, without being significant. In dyspnea, both groups obtained a significant improvement, the control group (*p* < 0.004) and the intervention group (*p* < 0.033).
(Alshaimaa et al., 2023) [15]	Impact Of Active Cycle Of Breathing Technique On Selected Pulmonary Outcomes In Post-COVID Syndrome Patients	Randomized controlled trial. *N* = 60. Participant age: 40–50 years. Intervention time: 12 weeks	**A.G**: aerobic exercise, muscle strengthening, and respiratory exercise. **B.G**: an active cycle of breathing technique in addition to what A.G received.	• Evaluating arterial blood gases. • 6-MWTD. • Fatigue: FAS.	In the 6-MWTD variable, a significant improvement was identified in both groups (*p* > 0.001). Both groups also obtained a reduction in fatigue, the difference between both groups being statistically significant (*p* < 0.001).
(Jimeno et al., 2022) [16]	Rehabilitation for post-COVID‐19 condition through a supervised exercise intervention: A randomized controlled trial	Randomized controlled trial. *N* = 39. Participant age: It does not indicate, > 18 years. Average age: 45.2 Intervention time: 8 weeks	**I.G**: tailored and supervised multicomponent exercise programme. **C.G**: WHO guidelines.	• Severity of symptoms And quality of life: SF-12. • Physical fitness: using a cycle exercise test. • Cardiopulmonary function: resting electrocardiogram and echocardiogram. In addition, FVC, FEV1, and MMV. • Dyspnea: MMRC. • Fatigue: FSS and CFS.	In comparison with the control group, supervised, controlled, and personalized training at low to moderate intensities has been found to be a more effective and safer intervention. Of the four variables relevant to our study (dyspnea, fatigue, pulmonary function, and quality of life), the latter three experienced significant improvement in the intervention group.
(Jimeno et al., 2023) [17]	Effects of a concurrent training, respiratory muscle exercise, and self-management recommendations on recovery from post-COVID-19 conditions: the RECOVE trial	Randomized controlled trial. *N* = 80. Participant age: It does not indicate, > 18 years. Intervention time: 8 weeks	**G.1 (CT)**: multicomponent exercise programme. **G.2 (RM)**: inspiratory muscle training programme. **G.3 (CTRM)**: multicomponent exercise programme plus inspiratory muscle training programme. **G.4 (CON)**: control group, informed to follow the WHO guideline.	• Cardiorespiratory fitness: submaximal multistage and individualized cardiopulmonary exercise test on a cycle ergometer. • Muscle strength: progressive loading test up to the 1RM. • Severity of symptoms: the 12-item Short Form Survey (SF-12). • Dyspnea: MMRC. • Fatigue: FSS and CFS.	G.1 and G.3 obtained better results compared to the other two groups (*p* < 0.05).

*Abbreviations:* *PImax* Maximum static inspiratory pressure, *SBP* Systolic blood pressure, *DBP* Diastolic blood pressure, *6-MWTD* 6-min walk test distance, *FSS* Fatigue Severity Scale, *MMRC* Modified Medical Research Council scale, *FEV*_*1*_ Forced expiratory volume for 1 second, *FVC* Forced vital capacity, *FEV*_*1*_ Forced expiratory volume at the end of the first second, *TLC* Total lung capacity, *FAS* Fatigue Assessment Scale, *MMV* Maximum voluntary ventilation, *CFS* the Chalder Fatigue Scale, *I.G* Intervention group, *C.G* Control group, *A.G* A Group, *B.G* B Group, *G.1* One group, *G.2* Two group, *G.3* Three group, *G.4* Four group

Table 3 is located here. It has been attached just before the bibliography since it would require the horizontal orientation of the page.

### Main results of the selected articles

In the first study, the intervention group underwent diaphragm release along with respiratory muscle training, while the control group only received inspiratory muscle training [13]. In the second study, the intervention group underwent pulmonary rehabilitation through virtual reality, whereas the control group received traditional pulmonary rehabilitation program (exercise on cycle ergometer, breathing exercises, general physical conditioning exercises, endurance training, and relaxation) [14]. In the third study, Group A received a traditional physiotherapy program including aerobic exercise, muscle strengthening, and respiratory exercise. Group B received an active breathing cycle (chest expansion exercises and forced expiration techniques) in addition to the traditional physiotherapy program [15]. In the fourth study, the intervention group underwent a personalized and supervised multicomponent exercise program, while the control group followed WHO guidelines [16]. Finally, in the fifth study, participants were divided into 4 groups: Group 1 underwent a multicomponent exercise program; Group 2 underwent inspiratory muscle training; Group 3 underwent both the multicomponent exercise program and inspiratory muscle training; and Group 4, the control group, followed WHO guidelines [17].

### Main outcomes

#### Exercise capacity with the 6-minute walk test (6-MWTD)

In one of the articles, this variable increased significantly in both groups, but there was a significantly greater improvement in the intervention group compared to the control group (*p* < 0.001) [13]. In another article, a significant improvement in this variable was identified in both the group that underwent pulmonary rehabilitation through virtual reality and the group that followed the conventional program. No significant difference in improvement was observed between the two groups [14]. In another article, a significant improvement was also identified in both groups after treatment (*p* > 0.001). Group B showed an improvement of 21.61%, while Group A achieved an increase of 11.09% [15].

### Lung function and maximum inspiratory pressure (PImax)

The first of the studies that assessed lung function did not demonstrate statistically significant improvement following the rehabilitation program [14]. Another article, which also examined lung function, showed significant improvement in the intervention group [16]. he last of the articles that assessed lung function did not identify significant changes in terms of VO2 max (*p* > = 0.05). However, significant individual improvements were observed in two of the groups, CT and CTRM (*p* < 0.05) [17] Regarding PImax, only one of the articles assessed it, showing a significant improvement in the intervention group (*p* < 0.001) and no change in the control group (*p* = 0.567), with a significant difference between both groups (*p* < 0.001) [13].

### Additional outcomes

#### Dyspnea

Regarding dyspnea, two of the articles evaluating this variable demonstrate a reduction in dyspnea in both groups, both in the control and intervention groups [13, 14]. In one of the studies, in the control group, there was a decrease in the mean difference of 12.81%, going from 2.42 (0.49) to 2.11 (0.33) (*p* < 0.01). As for the intervention group, there was a reduction of 48.89%, from 2.63 (0.60) to 1.38 (0.49) (*p* < 0.001) [13]. Therefore, despite significant improvement in both groups, the group that used diaphragm release plus inspiratory muscle training showed greater improvement compared to the group that only underwent inspiratory muscle training as a treatment. The scale used was mMRC [13]. In the second study, both groups demonstrated an improvement, as mentioned earlier. The improvement in the control group had a *p*-value of < 0.004, while in the intervention group, it was < 0.033. The comparison between both groups was not statistically significant. The scale used was The Borg scale [14].

On the other hand, another one of the articles assessing this variable showed that the control group exhibited partial improvement in dyspnea (*p* = 0.02) [16]. While both groups demonstrated similar improvements in symptoms, some of them disappeared more prominently in the intervention group, particularly dyspnea. The control group showed symptomatic improvement in the number of patients reporting dyspnea (controls versus exercise: 83.3% versus 5.4%, *p* = 0.003; V = 0.48) [16]. The population belonging to the intervention group reported a progressive improvement in symptoms after the intervention, being more likely to become asymptomatic (42.1% vs. 16.7%, *p* = 0.091). The scale used for the analysis was mMRC [16].

The last of the articles that studied this variable found a significant improvement (*p* < 0.05) in the CT group (multicomponent exercise program) and the CTRM group (multicomponent exercise program plus inspiratory muscle training program) compared to the control group (WHO recommendations) and the RM group (inspiratory muscle training). The scale used was mMRC [17].

#### Fatigue

Two of the studies evaluating this variable reveal a reduction in fatigue in both groups (control and intervention) [13, 15]. In the first study, fatigue was reduced from 43.36 ± 5.25 to 28.68 ± 6.01 (*p* < 0.001) in the intervention group and from 42.47 ± 5.18 to 39.77 ± 5.89 (*p* = 0.001) in the control group. There was a statistically significant difference between the two groups in favor of the intervention group (*p* < 0.001). The scale used for the analysis was the FSS [13]. In the other study, both groups showed a statistically significant difference after the intervention (*p* < 0.001). Group A had a reduction of 34.92%, while Group B experienced a decrease of 61.05%. The difference between both groups was statistically significant (*p* < 0.001). The scale used was the FAS [15].

Another study identified a significant improvement in the intervention group. The scales used were the FSS and CFS [16].

In the last of the articles, fatigue significantly improved in the CT and CTRM groups (*p* < 0.05). The scales used were the FSS and CFS [17].

### Quality of life

Two studies assessed the quality of life, and one of them identified a statistically significant improvement in the intervention group (*p* = 0.003) [16]. The other study did not achieve a statistically significant improvement in either of the groups [17].

## Discussion

This systematic review includes five randomized clinical trials that meet the inclusion and exclusion criteria outlined earlier, aiming to evaluate the effectiveness of pulmonary rehabilitation programmes and/or respiratory muscle training in patients with post-COVID conditions.

### Discussion about the results obtained

In the first study [13], four variables of relevance to our study were identified (6-minute walk test distance, dyspnea, fatigue, and PImax). In the first three variables, both groups improved after treatment, but in the 6-MWTD distance and fatigue, there was a statistically significant difference in favour of the intervention group. Regarding dyspnea, the improvement was also greater in the intervention group. As for PImax, significant improvement was only identified in the intervention group [13]. Hence, it could be concluded that while both groups showed improvement in most of the examined variables, the treatment combining diaphragm release with inspiratory muscle training was more effective than the treatment consisting solely of inspiratory muscle training [13].

In the second of the articles included in this review [14], two important variables for our study were assessed (6-MWTD and dyspnea). Regarding the first variable, there was improvement in both groups without a significant difference. However, concerning dyspnea, although there was improvement in both groups, the control group showed a greater improvement. Although not as clear in this case, it could be argued that the traditional pulmonary rehabilitation programme (including cycle ergometer exercises, breathing exercises, general fitness exercises, resistance training, and relaxation) was more effective than pulmonary rehabilitation using virtual reality [14].

The third study [15], examined two variables, the 6-MWTD and fatigue. Both variables improved after treatment in both groups, with group B showing better results in the 6-MWTD. Regarding fatigue, group B also demonstrated a statistically significant improvement compared to group A [15]. Taking into account these variables, it could be said that the group receiving the traditional physiotherapy programme (aerobic exercise, muscle strengthening exercises, and respiratory exercises) along with the Active Breathing Cycle technique (based on a cycle for controlling breathing, including chest expansion exercises and forced expiration techniques to clear bronchial secretions and promote increased lung volume) was more effective than the group that only received the traditional physiotherapy programme [15].

The fourth article [16], identified four relevant variables for this study (dyspnea, fatigue, lung function, and quality of life). In the last three variables, the intervention group experienced a significant improvement. Regarding dyspnea, both groups benefited, with the intervention group showing a more pronounced improvement [16]. Hence, it is evident that a multicomponent exercise programme (combining resistance training with aerobic training) was more effective than following the WHO guidelines alone [16].

To conclude with, the last study [17], assessed four variables dyspnea, fatigue, lung function, and quality of life. The first two showed significant positive changes in two of the groups (CT and CTRM). Regarding lung function, both of the aforementioned groups did not exhibit a significant overall improvement, but significant individual improvements were identified. Lastly, quality of life did not show statistically significant changes in any of the four study groups. Therefore, it is clear that a treatment that includes a multicomponent exercise programme or this programme combined with inspiratory muscle training was more effective than inspiratory muscle training alone or following WHO recommendations [17].

### Pulmonary rehabilitation programmes and/or respiratory muscle training

In conclusion, considering the results obtained from the various included studies, it becomes evident that the most effective treatment approach involves combining a personalized and supervised pulmonary rehabilitation programme (aerobic training and strength training) along with inspiratory muscle exercises, as separately they have not achieved such significant results.

Other systematic reviews are in line with the results obtained in this study [19].

Rehabilitation programmes consisting of aerobic exercise, anaerobic exercise, and respiratory training could be the key treatment to alleviate post-COVID symptoms such as fatigue, dyspnea, reduced respiratory function, physical condition, and quality of life [19]. A prospective study [20], assessed the effects of a treatment programme comprising interval training, muscle strength exercises, and individualized respiratory exercises in 39 individuals with post-COVID sequelae. The study concluded that a personalized treatment programme containing the aforementioned elements demonstrated positive effects on dyspnea, aerobic endurance, and cardiorespiratory performance [20]. Furthermore, it is important to highlight that after the two-year follow-up, a reduction in dyspnea was achieved in all participants in the study. At the two-year mark, none of the participants exhibited any pre- or post-treatment side effects or adverse effects [20]. In an observational cohort study, 58 patients with respiratory sequelae underwent a 6-week individualized rehabilitation programme, which included resistance training, strength training, and inspiratory muscle training. The study supported that a comprehensive and personalized rehabilitation programme improved the fatigue and functional limitations experienced by the participants [21]. Another systematic review, which included 20 articles, also concluded that aerobic training, along with muscle strengthening exercises and inspiratory muscle training techniques, could be an effective treatment option for patients with post-COVID symptoms [22].

### Scales used for dyspnea, fatigue and quality of life

Firstly, regarding the dyspnea variable, of the 4 included studies that assess this variable, 3 measure dyspnea using the Modified Medical Research Council scale (mMRC) [13, 16, 17]. The other study measured this variable using the Borg scales [14]. Although there is no clear guideline on which scale to use for patients with post-COVID conditions, most studies utilise these two scales. Another article that was found also used the Borg scales [23], but a greater number of studies employ the mMRC scale [20, 24–27].

Regarding fatigue, of the 4 articles that examine this variable, three of them use the Fatigue Severity Scale (FSS) [13, 16, 17]. The other article used the Fatigue Assessment Scale (FAS) [15]. Due to the lack of consensus on a specific scale for assessing fatigue in post-COVID patients, there is a variety of scales chosen by different studies to measure this variable. Two of the studies found use the FAS [21, 28], In contrast, another study uses the FSS [29]. However, another study found uses a different scale than the ones mentioned previously (FACIT-Fatigue) [30].

Regarding quality of life, the two articles that studied this variable used the 12-item Short Form Survey (SF-12) [16, 17]. There is also no consensus on which quality of life scale is most suitable to use in this population. Each article employs different scales. For instance, one study also uses the SF-12 [31]. However, other studies use various questionnaires, for example, the Short-Form 36 Questionnaire (SF-36) [20], EuroQol visual analogue scale (VAS) [32], the EuroQol Group 5-dimension 5-level (EQ-5D-5 L) questionnaire [21], the Euro-QoL-5D (EQ-5D) questionnaire [33].

One thing that is clear is that a significant percentage of articles studying different treatments in this population use the 6-MWTD to assess physical capacity [20, 21, 23, 33–39].

Taking into account the aforementioned, we can observe that there is no clear consensus regarding which scales or tests to use for the different variables evaluated in patients with post-COVID conditions, although there are some that are more commonly used than others.

### Limitations

As the main limitation of the article, there was a limited number of clinical trials that met the inclusion and exclusion criteria. Many of the articles found were excluded because they were conducted remotely or through virtual reality. Therefore, further research is needed in individuals with post-COVID condition to evaluate the effectiveness of an in-person, individualized program that includes both aerobic and muscular training, as well as inspiratory muscle training.

## Conclusions

Despite the lack of a specific treatment at present and considering the scarcity of studies that specifically assess treatment effectiveness, it is evident from this review that a well-designed pulmonary rehabilitation programme comprising aerobic exercise, muscular strength exercises, and inspiratory muscle training techniques and exercises showed significant efficacy.

The previously mentioned treatment leads to significant improvements in the main post-COVID sequelae, including fatigue, dyspnea, lung function, physical capacity, and consequently, quality of life.

## Data Availability

The conducted searches, as well as the number of articles found, examined, and excluded, can be replicated by following Appendix 2: Search Strategy, and in Fig. 1: Flowchart. In addition, the different searches conducted for each journal are described in these sections.

## Appendix 1

**Table:** PRISMA checklist

Section/topic	#	Checklist item		Reported on page #
*TITLE*				
Title	1	Identify the report as a systematic review, meta-analysis, or both.		YES (page 1)
*ABSTRACT*				
Structured summary	2	Provide a structured summary including, as applicable: background; objectives; data sources; study eligibility criteria, participants, and interventions; study appraisal and synthesis methods; results; limitations; conclusions and implications of key findings; systematic review registration number.		YES (page 1)
*INTRODUCTION*				
Rationale	3	Describe the rationale for the review in the context of what is already known.		YES (page 2)
Objectives	4	Provide an explicit statement of questions being addressed with reference to participants, interventions, comparisons, outcomes, and study design (PICOS).		YES (page 3)
*METHODS*				
Protocol and registration	5	Indicate if a review protocol exists, if and where it can be accessed (e.g., Web address), and, if available, provide registration information including registration number.		YES (page 3)
Eligibility criteria	6	Specify study characteristics (e.g., PICOS, length of follow-up) and report characteristics (e.g., years considered, language, publication status) used as criteria for eligibility, giving rationale.		YES (pages 4)
Information sources	7	Describe all information sources (e.g., databases with dates of coverage, contact with study authors to identify additional studies) in the search and date last searched.		YES (pages 3)
Search	8	Present full electronic search strategy for at least one database, including any limits used, such that it could be repeated.		YES (page 3–4)
Study selection	9	State the process for selecting studies (i.e., screening, eligibility, included in systematic review, and, if applicable, included in the meta-analysis).		YES (page 6)
Data collection process	10	Describe method of data extraction from reports (e.g., piloted forms, independently, in duplicate) and any processes for obtaining and confirming data from investigators.		YES (page 4)
Data items	11	List and define all variables for which data were sought (e.g., PICOS, funding sources) and any assumptions and simplifications made.		YES (page 3)
Risk of bias in individual studies	12	Describe methods used for assessing risk of bias of individual studies (including specification of whether this was done at the study or outcome level), and how this information is to be used in any data synthesis.		YES (page 5)
Summary measures	13	State the principal summary measures (e.g., risk ratio, difference in means).		Yes (page 5)
Synthesis of results	14	Describe the methods of handling data and combining results of studies, if done, including measures of consistency (e.g., I^2^) for each meta-analysis.		NO
**Section/topic**	**#**	**Checklist item**	**Reported on page #**	
Risk of bias across studies	15	Specify any assessment of risk of bias that may affect the cumulative evidence (e.g., publication bias, selective reporting within studies).	NO	
Additional analyses	16	Describe methods of additional analyses (e.g., sensitivity or subgroup analyses, meta-regression), if done, indicating which were pre-specified.	NO	
*RESULTS*				
Study selection	17	Give numbers of studies screened, assessed for eligibility, and included in the review, with reasons for exclusions at each stage, ideally with a flow diagram.	YES (page 5–6)	
Study characteristics	18	For each study, present characteristics for which data were extracted (e.g., study size, PICOS, follow-up period) and provide the citations.	YES (pages 8, 9, 10 and 11)	
Risk of bias within studies	19	Present data on risk of bias of each study and, if available, any outcome level assessment (see item 12).	YES (page 7)	
Results of individual studies	20	For all outcomes considered (benefits or harms), present, for each study: (a) simple summary data for each intervention group (b) effect estimates and confidence intervals, ideally with a forest plot.	YES (page 12,13 and 14)	
Synthesis of results	21	Present results of each meta-analysis done, including confidence intervals and measures of consistency.	NO	
Risk of bias across studies	22	Present results of any assessment of risk of bias across studies (see Item 15).	YES (page 7)	
Additional analysis	23	Give results of additional analyses, if done (e.g., sensitivity or subgroup analyses, meta-regression [see Item 16].	NO	
*DISCUSSION*				
Summary of evidence	24	Summarize the main findings including the strength of evidence for each main outcome; consider their relevance to key groups (e.g., healthcare providers, users, and policy makers).	YES (pages 14, 15, 16)	
Limitations	25	Discuss limitations at study and outcome level (e.g., risk of bias), and at review-level (e.g., incomplete retrieval of identified research, reporting bias).	YES (page 17)	
Conclusions	26	Provide a general interpretation of the results in the context of other evidence, and implications for future research.	YES (page 17)	
*FUNDING*				
Funding	27	Describe sources of funding for the systematic review and other support (e.g., supply of data); role of funders for the systematic review.	YES (page 18)	

*From*: Moher D, Liberati A, Tetzlaff J, Altman DG, The PRISMA Group (2009). Preferred Reporting Items for Systematic Reviews and Meta-Analyses: The PRISMA Statement. PLoS Med 6 (7): e1000097. 10.1371/journal.pmed1000097.

## Appendix 2

**Table:** Search strategy

Nº term used	
**#1** Respiratory muscle training **#2** Inspiratory muscle training **#3** Expiratory muscle training **#4** Maximum inspiratory pressure **#5** Maximum expiratory pressure **#6** Breathing training **#7** Respiratory rehabilitation **#8** Pulmonary rehabilitation **#9** Post COVID syndrome **#10** Patients with COVID sequelae **#11** Long COVID patients	

### PubMed

#### 8 Potential articles

((“respiratory muscle training“[Title/Abstract]) OR (“inspiratory muscle training“[Title/Abstract]) OR (“expiratory muscle training“[Title/Abstract]) OR (“maximum inspiratory pressure“[Title/Abstract]) OR (“maximum expiratory pressure“[Title/Abstract])) AND ((“long COVID“[Title/Abstract]) OR (“post COVID syndrome“[Title/Abstract]))

#### 66 potential articles

(breathing training) AND (long-covid patients).

#### 576 potential articles

(respiratory rehabilitation) AND (patients with covid sequelae).

### PEDro

#### 4 Potential articles

(pulmonary rehabilitation) AND (patients with sequelae of covid-19).

### Embase

#### 10 potential articles

(‘breathing’ OR ‘breathing’/exp OR breathing) AND (‘training’ OR ‘training’/exp OR training) AND (‘patients’ OR ‘patients’/exp OR patients) AND with AND ‘post covid’ AND sequelae.

#### 36 potential articles

(‘pulmonary rehabilitation’/exp OR ‘pulmonary rehabilitation’) AND (‘patients’/exp OR patients) AND with AND ‘post covid’ AND sequelae.

### Cochrane

#### 13 potential articles

(Breathing training) AND (long-covid patients).

#### 14 potential articles

(pulmonary rehabilitation) AND (patients with sequelae of Covid-19).

### Scopus

#### 25 potential articles

(Breathing training) AND (long-covid patients).

#### 75 potential articles

(pulmonary rehabilitation) AND (patients with sequelae of covid-19).

### Web of Science

#### 4 Potential articles

(Breathing training) AND (long-covid patients).

#### 97 potential articles

(pulmonary rehabilitation) AND (patients with sequelae of covid-19).

#### 74 potential articles

(pulmonary rehabilitation) AND (long-covid patients).

## Abbreviations

SARS-CoV-2: Severe acute respiratory syndrome coronavirus-2
WHO: World health organization
PRISMA: Preferred reporting items for systematic reviews and meta-analysis
PROSPERO: International prospective register of systematic reviews
PICO: (P): population; (I): intervention; (C): comparison; (O): outcome
PEDro: Physiotherapy evidence database
PImax: Maximum static inspiratory pressure
SBP: Systolic blood pressure
DBP: Diastolic blood pressure
6-MWTD: 6-min walk test distance
FSS: Fatigue Severity Scale
MMRC: Modified medical research council scale
FEV_1_: Forced expiratory volume for 1 second
FVC: Forced vital capacity
TLC: Total lung capacity
PSS-10: The perceived stress scale
FAS: Fatigue assessment Scale
MMV: Maximum voluntary ventilation
CFS: Chalder Fatigue Scale
SF-12: 12-item short form survey
CT: Multicomponent exercise programme
CTRM: Multicomponent exercise programme plus inspiratory muscle training programme
RM: Inspiratory muscle training
